# Disclosure of intimate partner violence by men and women in Dar es Salaam, Tanzania

**DOI:** 10.3389/fpubh.2022.928469

**Published:** 2022-09-26

**Authors:** Enryka Christopher, Ndeye D. Drame, Germana H. Leyna, Japhet Killewo, Till Bärnighausen, Julia K. Rohr

**Affiliations:** ^1^Harvard Center for Population and Development Studies, Harvard T.H. Chan School of Public Health, Cambridge, MA, United States; ^2^Department of Epidemiology, Muhimbili University of Health and Allied Sciences, Dar es Salaam, Tanzania; ^3^Heidelberg Institute of Global Health, Faculty of Medicine, University of Heidelberg, Heidelberg, Germany

**Keywords:** violence, Intimate Partner Violence (IPV), Africa, disclosure, list experiment, methodology, sexual abuse, physical abuse

## Abstract

Intimate Partner Violence (IPV) has severe health consequences, though may be underreported due to stigma. In Tanzania, estimates of IPV prevalence range from 12 to >60%. List experiments, a technique of indirectly asking survey questions, may allow for more accurate prevalence estimates of sensitive topics. We examined list experiment and direct questions about experiences of physical and sexual IPV from a 2017 cross-sectional survey among 2,299 adults aged 40+ years in Dar es Salaam. List experiment prevalence estimates were determined through quantitative analysis and compared qualitatively to direct question prevalence estimates. The list experiment estimated a higher prevalence of IPV in all cases except for physical violence experienced by women. This study contributes to the estimation of IPV prevalence. If the list experiment estimates yield an unbiased estimate, findings suggest women openly report experiencing physical IPV, and IPV experienced by men is underreported and understudied.

## Introduction

Intimate Partner Violence (IPV) encompasses “any behavior within an intimate relationship that causes physical, psychological or sexual harm to those in the relationship” and causes large disease burdens globally (1). IPV is well documented to have severe psychological and physical consequences for the victims (1–8). Research over the past several decades have garnered various prevalence rates of IPV from around the globe (8–11), with the prevalence rates of IPV in African countries being among the highest in the world (8, 9). However, underreporting of IPV is a common challenge faced by researchers studying this public health concern and little is known about IPV and its consequences in African countries (12–15).

Tanzania is an east African country with estimates of IPV prevalence ranging between 12.3 and 60%, with rates varying between urban and rural contexts and for different populations (9, 16–20). However, these prevalence rates are likely to be underestimates because a number of studies have suggested that victims of IPV in Tanzania face barriers not only to reporting their abuse to health or law enforcement officials but also disclosing the information to anyone at all. Stigma and shame surrounding a culture of IPV, lack of available resources to guide reporting of IPV incidences, and fear of criminal prosecution for men who have sex with men under Tanzania's strict anti-homosexuality laws are some of the many reasons why victims often don't disclose their experience (21–28). These barriers are even more pronounced with men who experience IPV, with very little known about men who encounter physical or sexual abuse as the majority of research in this field focuses on IPV against women (29, 30). Two studies that have looked into prevalence rates of males experiencing IPV suggest that quite a large percentage of men in Tanzania have been physically abused, with prevalence estimates of 19 and 34% reported (29, 30).

Underreporting of IPV is likely common because sociocultural norms of Tanzania tolerate violence against women, thus victims who speak out often face shame and stigma from the community (21). Additionally, research suggests that alcohol use is commonly involved in IPV incidents, adding both another layer of stigma that surrounds substance abuse as well as potentially altering the victim's state of mind during the violence, making their memory of the incident weak (31, 32). Even if these social stigmas did not exist, Tanzanian healthcare workers have limited resources to give support, leading to a reluctance in reporting to an overburdened system ineffective in aiding victims' recovery (33). Furthermore, men who experience IPV from male partners likely report to an even lesser extent, given the stigma they may face in Tanzania's heavily gendered society. Men who have male partners may also fear criminal prosecution, as homosexual acts are punishable by hefty fines and long prison sentences (28).

One study examining IPV experienced by ever married Tanzanian women found that around 30% of women aged 35–49 in their Tanzanian sample experienced IPV (this number was much higher for young women) (34). Another, more recent study using Tanzanian DHS data, found an IPV prevalence rate of around 31% (35). A study in Pakistan, a similar developing economy context to Tanzania, found around 31% of women in their sample reported emotional IPV and 18% reported experiencing physical violence (36). None of these studies, however, included men in their sample, differentiated between sexual and physical IPV.

Methods have been rapidly evolving to assess sensitive and stigmatizing experiences, and list experiments are among the most promising approaches. List experiments are a technique of indirectly asking questions in surveys. They may be a key to finding the prevalence of IPV more accurately because the participant does not directly disclose their answers to the interviewer, lowering social desirability bias (37–39). However, efficacy of the list experiment is variable, and more research is needed to understand contexts in which this method works best (39–41). We use cross-sectional survey data from the “Health and Aging in Africa: A Longitudinal Study in three INDEPTH Communities” (HAALSI) survey in Tanzania to examine the potential for bias in reporting of IPV by adult men and women over 40 years of age in Dar es Salaam. We use list experiments to achieve the following scientific aims: to (1) to estimate the prevalence of sexual and physical IPV in men and women through a list experiment and through directly asked questions, and (2) measure the extent to which physical and sexual IPV is underreported when the traditional direct survey questions to elicit IPV data are used. Based on the stigma barriers and the sensitivity of the topic, we hypothesized that there would be a substantial increase in IPV disclosure through the list experiment compared to the direct questions for all groups in our study- women experiencing sexual violence, women experiencing physical violence, men experiencing sexual violence, and men experiencing physical violence.

The following study contributes useful knowledge about IPV in the Tanzanian urban context, as available research has not included men, differentiated between sexual and physical IPV, or trialed survey methods to measure or test reporting bias on this topic. It is imperative to understand the extent of IPV that man experience, to indicate whether this reversal of traditionally gendered power and abuse dynamics is widespread, and if so, to develop support services that meet the needs of male IPV victims. This study is also a relevant addition to the growing body of knowledge on list experiments.

## Methods

HAALSI is a family of aging studies nested within existing health and demographic surveillance systems (HDSS) in Africa. Comparable to the U.S. Health and Retirement Study (HRS) and other international sister studies, HAALSI is adapted to address specific characteristics of older adult populations of Sub-Saharan Africa in order to understand the behavioral and biological risks that determine healthy aging in these countries. The baseline HAALSI surveys were conducted in South Africa (2015), Ghana (2016), and Tanzania (2017). The HAALSI Tanzania survey included adults aged 40 years and older living in the Ukonga and Gongo la Mboto wards of Ilala district in Dar es Salaam, Tanzania. The study sample was embedded within the Dar es Salaam Urban Cohort Study (DUCS) in partnership with Muhimbili University of Health and Allied Sciences. Data collection methods were adapted from the HAALSI South Africa survey (42). The 2013 HDSS census data was used as a sampling frame, from which the researchers randomly selected participants to include in the study. Field researchers then visited each participant's home to obtain informed consent from the participant, with those unable to read using a witness and an inked fingerprint as signature. Field researchers administered in-person interviews in the Kiswahili language, with responses captured on tablet computers.

Key variables included age, marital status, education, and employment in addition to sexual and physical IPV, the variables of interest. Marital status included “Never Married,” “Separated/Divorced,” “Widowed,” and “Currently Married/Cohabiting.” Education included all levels from none to university level education as well as “Vocational training.” Employment status could be “Employed,” “Unemployed,” or “Homemaker.” The options for these key variables were formed based on the research team's demographic knowledge of the population.

Within the HAALSI Tanzania baseline survey, a list experiment component was included in the HAALSI Tanzania survey alongside direct questions about IPV to demonstrate whether a different depiction of the prevalence of IPV disclosure would be observed in the sample. List experiments, or “item count technique,” is a form of indirect questioning that has been used in social sciences to reduce the impact of social desirability bias, especially when asking sensitive questions (39, 40). Participants are randomly divided into Group A and Group B, with both groups being given a list of statements (e.g., “I drink soda every day,” “I have diabetes,” etc.) and asked to keep a count of how many are true to them. Group A received 4 items in the list, while Group B received the same 4 Group A items plus a fifth item addressing the sensitive IPV topic. The difference in the average number of items counted between these two groups aid in understanding the unbiased prevalence of the sensitive item. Participants are more likely to disclose truthful answers through this method because the interviewer is unable to determine which of the items pertain to the individual and this limits any concerns of stigma the participants may have.

We first found the prevalence of the direct IPV questions through a proportion calculation stratified by sex, with 95% confidence intervals. The list experiment prevalence was then calculated by taking the difference in mean response between Group A (4-item) and Group B (5-item). Confidence intervals for the list experiment estimates was calculated using R package “List: Statistical Methods for the Item Count Technique and List Experiment” (43). We compared the prevalence and 95% confidence intervals of physical and sexual IPV experienced by men and women derived from the list experiments and direct questions to highlight any differences in reporting based on questioning methods.

## Results

The survey was conducted among 2,299 individuals, including 32.4 male and 67.6% female. The majority of the study population was married or cohabitating (69.4%), had received at least standard level education (82.6), and 46% were employed (Table 1).

**Table 1:** Demographic characteristics of the HAALSI Tanzania cohort, stratified by sex.

	**All (*N* = 2299)**	**Female *n* = 1555 (67.64%)**	**Male *n* = 744 (32.36%)**
**Mean age (SD)**	52.96 (11.1)	51.78 (10.8)	55.45 (11.2)
**Age**			
40–49	1108 (48.2%)	822 (52.9%)	286 (38.4%)
50–59	615 (26.8%)	410 (26.4%)	205 (27.6%)
60–69	379 (16.5%)	216 (13.9%)	163 (21.9%)
70–79	135 (5.9%)	64 (4.1%)	71 (9.5%)
80+	62 (2.7%)	43 (2.8%)	19 (2.6%)
**Marital status**			
Never married	70 (3.0%)	58 (3.7%)	12 (1.6%)
Separated/divorced	196 (8.5%)	161 (10.4%)	35 (4.7%)
Widowed	395 (17.2%)	352 (22.6%)	43 (5.8%)
Married/cohabiting	1596 (69.4%)	963 (61.9%)	633 (85.1%)
Missing	42 (1.8%)	21 (1.4%)	21 (2.8%)
**Highest education level**			
None	358 (15.6%)	305 (19.6%)	53 (7.1%)
Standard levels 1–7	1393 (60.6%)	954 (61.4%)	439 (59.0%)
Form 1–6	409 (17.8%)	235 (15.1%)	174 (23.3%)
University level +	60 (2.6%)	25 (1.6%)	35 (4.7%)
Vocational training	35 (1.5%)	13 (0.8%)	22 (3.0%)
Missing	44 (1.9%)	23 (1.48%)	21 (2.8%)
**Employment status**			
Employed	1035 (45.0%)	625 (40.9%)	410 (56.8%)
Unemployed	439 (19.1%)	227 (14.9%)	212 (29.4%)
Homemaker	763 (33.2%)	672 (44.0%)	91 (12.6%)
Missing	62 (2.7%)	31 (2.0%)	31 (4.17%)

**Figure 1:**
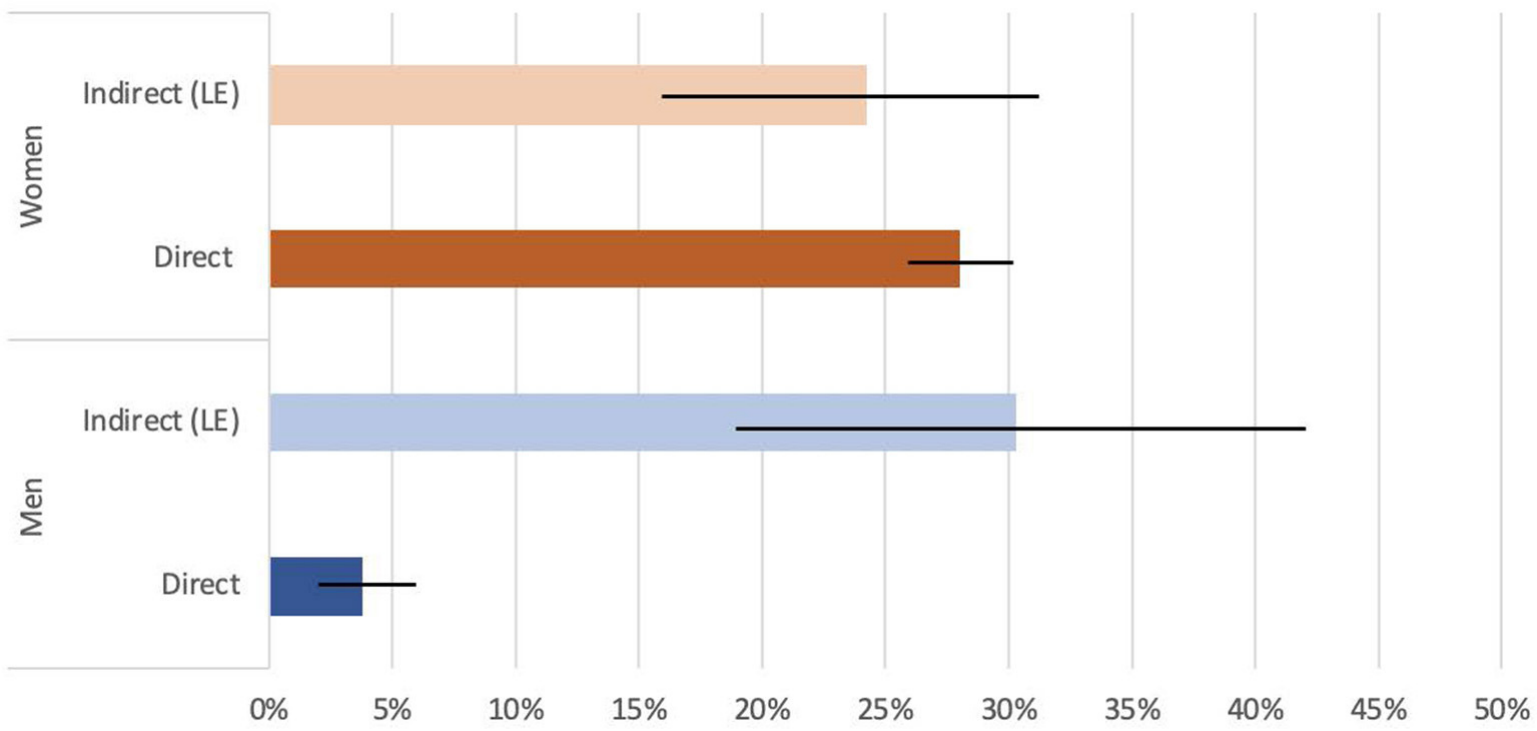
Direct and indirect (List Experiment) physical IPV disclosure, stratified by sex.

**Figure 2:**
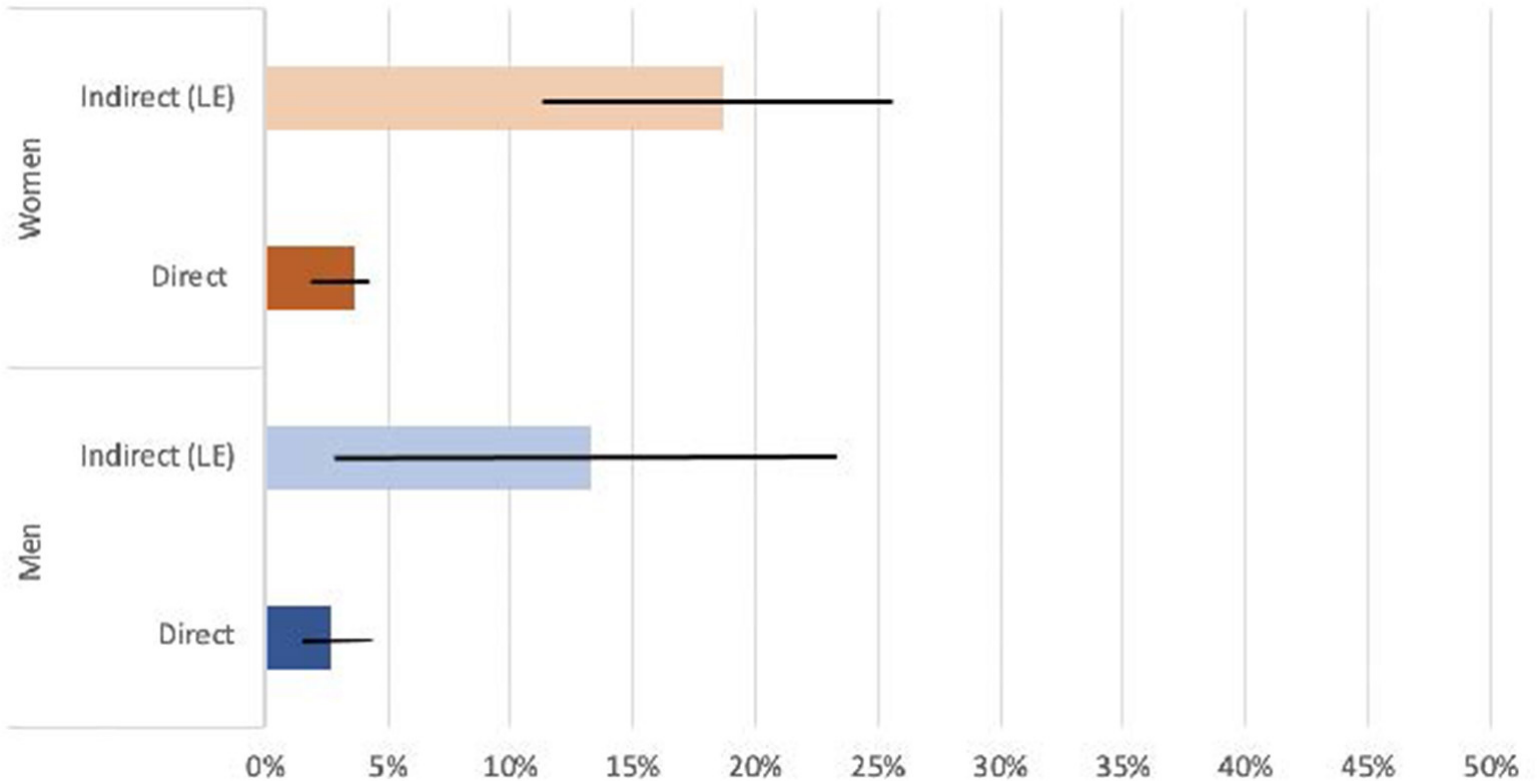
Direct and indirect (List Experiment) sexual IPV disclosure, stratified by sex.

The list experiment estimated a higher prevalence of IPV experience reporting in all cases except for physical violence experienced by women. Men disclosed more physical IPV in the list experiment [30.3%, 95% CI (19.2, 41.4)] than in the direct question [3.8%, 95% CI (2.4, 5.2)] and more sexual IPV in the list experiment [13.3%, 95% CI (2.8, 23.8)] than in the direct question [2.7%, 95% CI (1.5, 3.9)]. Report of physical IPV among women was similar for the list experiment [24.2%, 95% CI (16.7, 31.7)] and the direct question [28.0%, 95% CI (25.7, 30.3)]. Yet, women disclosed more sexual IPV in the list experiment [18.7%, 95%CI (11.7, 25.7)] than in the direct question [3.6%, 95% CI (2.6, 4.6)] (see Figures 1, 2).

## Discussion

For physical IPV reported by women, the prevalence estimates obtained through the list experiment (24.2%) and the direct question (28.0%) were quite similar. The prevalence estimate for physical IPV among women found in this study mirrors the prevalence for physical IPV experienced by women found in the Demographic and Health Survey (DHS) of Tanzania, in which data on physical and sexual IPV were collected from women. In fact, 21% of women in the DHS reported ever experiencing at least less than severe physical violence by a husband or partner (44). When asked about partner violence, more than 37% of men in the DHS responded that it was acceptable to beat their wives or partners in at least one hypothetical situation (44). This normalization of physical violence toward women within a relationship could explain why the direct IPV question in our study resulted in such a similar prevalence estimate to the list experiment and was even higher than the list experiment result; because wife or partner beating is condoned in Tanzania by many men and socially accepted to a certain extent, women may be less reluctant to disclose their experience of physical IPV.

There was, however, a difference in prevalence estimates for sexual IPV reported by women. A higher percentage of women reported sexual violence in the list experiment (18.7%) rather than in the direct questions (3.6%); these prevalence estimates differ from those found in the DHS data (12.4%). Surprisingly, considerably less men said it was acceptable to beat their wives for refusing to have sex (13.1%) than other reasons (44). This could mean that sexual violence between partners is more taboo or stigmatized, and thus those women who do experience it are less likely to disclose their sexual IPV experience when asked directly. If this is the case, and the list experiment provides accurate estimates, list experiments may obtain more accurate results when asking women about experiences of sexual IPV in surveys.

For physical violence experienced by men, there was quite a large gap between the prevalence estimates from the list experiment and the direct question. The prevalence gathered by our direct question was very low (3.8%) compared to that of our list experiment (30.3%). Our list experiment prevalence estimate is similar to the prevalence reported in two studies that looked at physical IPV experienced by men (29, 30). Thus, if the list experiment provides accurate estimates, this disparity may imply that list experiments may gauge the prevalence of physical IPV experienced by men better than direct questioning would.

For sexual violence experienced by men, the list experiment prevalence estimate (13.3%) was higher than the direct question prevalence estimate (2.7%), although the confidence intervals overlap. As there is no other research on sexual IPV experienced by men in Tanzania, we do not have any other prevalence estimates from this specific context to compare ours with. One study was found that looked at sexual IPV experienced by gay men among six countries, including South Africa; this study found a prevalence rate of around 4% (45). The prevalence estimate of male sexual IPV victimization could be higher than the 13.3% reported by the list experiment, as it is possible that some men would not have reported their experience in either the list experiment or direct question because of the highly stigmatized nature of sexual violence against men.

In other Tanzanian studies on IPV, researchers reported that around 30% of women aged 35–49 in one study experienced IPV, and 31% from a sample from the Demographic and Health Survey data (34, 35). Compared to this finding, our prevalence estimates of 28% for physical IPV reported by the women in our sample through direct questions is similar, but the prevalence estimate of 24% obtained through the list experiment is slightly lower. Our study's findings of 4 and 19% in the direct questions and list experiment for sexual IPV, respectively, are also lower than the IPV prevalence reported by this study.

We also compared findings to the study in Pakistan (36), finding that compared to those who reported emotional IPV in Pakistan (31%), our estimate for physical IPV obtained through direct questions is similar (28%), but our estimate obtained through the list experiment is slightly lower (24%). Compared to those who reported emotional IPV in Pakistan (31%), our study's findings of 4% in the direct questions and 19% in the list experiment for sexual IPV are both much lower. Compared to those who reported physical violence in Pakistan (18%), our estimates for physical IPV obtained through direct questions (28%) and the list experiment (24%) are both somewhat higher. Compared to those who reported physical violence in Pakistan (18%), our study's estimate for sexual IPV obtained through direct questioning was much lower (4%). However, our study's estimate for sexual IPV obtained through list experiment (19%) was similar the percentage of those who reported physical violence in Pakistan (18%).

This study provides important contributions to the estimation of IPV prevalence in urban Tanzania. We provide evidence that suggests list experiment methods may effectively account for reporting bias in this context, as well as other east African countries or other cultures that have similar stigma surrounding IPV. If the list experiment estimates are accurate, the direct survey questions underestimate experience of sexual IPV by women and both physical and sexual IPV experienced by men, which would suggest that IPV experienced by men is widespread and understudied. An enormous strength of this study is that this is the first study to look at male victims of sexual IPV in Tanzania, setting the groundwork for future studies to build upon. Additionally, because there has been a wide range of results from studies on list experiments, studies such as the current one are important additions to the literature on this survey technique in order to build our knowledge of when using list experiments are most appropriate.

## Limitations

We would like to acknowledge several limitations of this study. One study on female attitudes toward IPV revealed that women were much more likely to justify IPV when other women were present during an interview than in the presence of a man (15). Similarly, the DHS dataset revealed that when asked if it was acceptable to beat a wife, men who were in the presence of their wives were more likely to say yes than if they were asked alone. These data suggest that the results of this study may be influenced by the gender of our interviewers, as we randomized both male and female interviewers to different participants. Attitudes toward IPV provide significant anthropological insight for context, thus it was unfortunate that participants were not asked questions about their views on IPV in the HAALSI Tanzania survey. Having this data may have provided a fuller scope and deeper understanding of our population.

The population that participated in the HAALSI Tanzania survey were 40 years of age and older, while the majority of research on IPV focuses on younger age groups. While this study does fill an important gap, our findings may not be generalizable to younger populations or comparable to studies that use younger cohorts.

Although the inclusion of a list experiment is a strength of this study, it can also be a limitation because analyses done with this survey technique may be prone to error. There is some contention among the research community on the efficacy of list experiments (46). Therefore, we would like to emphasize that the list experiment results of this study highlights the discrepancy between those who report in list experiments and direct questions, rather than providing an exact estimate of IPV prevalence. After speaking to an expert statistician, our team ultimately decided to keep our findings closer to qualitative methodology rather than quantitative. We view these findings to indicate that this topic may be a trend that merits further research on a larger sample size.

Finally, the phrasing of both the direct IPV questions and list experiment statement were quite direct, but vague; “Have you ever been raped by your partner?” and “I have ever experienced physical violence committed by my partner” were the formats of the included questions and statements in the HAALSI Tanzania survey. In a culture that normalizes violence and does not recognize marital rape (47), participants may not have understood what IPV encompasses. More detailed questions would have benefitted this dataset.

## Future directions

More research is needed to understand the scope of IPV faced by women. However, the lack of research on men experiencing IPV presents an urgent need for more studies that focus on not only estimating prevalence of IPV in different populations of men across Tanzania, but also the health consequences male victims of IPV face. Understanding the true prevalence of physical and sexual IPV is the first step toward providing support to victims.

The results of this study suggest that list experiment methodology may work in this setting, although more research is needed to understand its accuracy. Future research should continue to test list experiments in various cultures in order to extend our knowledge on what sort of stigmatized issues would benefit from implementing this survey technique.

In future studies seeking to estimate prevalence of IPV, it is imperative that the participants are asked about attitudes toward IPV as well so that the IPV prevalence of that particular population can be contextualized more thoroughly.

## Data availability statement

The raw data supporting the conclusions of this article are available by request from the authors, without undue reservation.

## Ethics statement

The studies involving human participants were reviewed and approved by the ethics review board of the Muhimbili University of Health and Allied Sciences. All patients/participants provided their written informed consent to participate in this study.

## Author contributions

JK, GL, JR, and TB conceptualized the study and provided review and feedback. EC and ND did analysis and wrote full manuscript. All authors contributed to the article and approved the submitted version.

## Funding

Research reported in this publication was supported by the National Institute on Aging of the National Institutes of Health under Award Number P30AG024409.

## Conflict of interest

The authors declare that the research was conducted in the absence of any commercial or financial relationships that could be construed as a potential conflict of interest. The reviewer SK declared a shared affiliation with the authors GL and JK to the handling editor at the time of review.

## Publisher's note

All claims expressed in this article are solely those of the authors and do not necessarily represent those of their affiliated organizations, or those of the publisher, the editors and the reviewers. Any product that may be evaluated in this article, or claim that may be made by its manufacturer, is not guaranteed or endorsed by the publisher.

## Author disclaimer

The content is solely the responsibility of the authors and does not necessarily represent the official views of the National Institutes of Health.
