# Supplementary material for: Disclosure of intimate partner violence by men and women in Dar es Salaam, Tanzania
Source: Front Public Health. 2022 Sep 26;10:928469. doi: 10.3389/fpubh.2022.928469 (PMC9549336; doi:10.3389/fpubh.2022.928469)
Supplement: Supplementary file 1 [file Data_Sheet_1.DOCX]

**Intimate Partner Violence in Tanzania**

**Supplementary Analysis**

Table 1. Logistic regression analysis with key variables – Physical violence

|  |  |  |  |  |
| --- | --- | --- | --- | --- |
| **Physical violence** | | | | |
| Characteristics | OR | Standard Error | 95% CI | P-value |
| **Sex** |  |  |  |  |
| Male | REF. | - | - | - |
| Female | 1.19 | 0.37 | 0.64-2.20 | 0.57 |
| **Age group** |  |  |  |  |
| 40-49 | REF. | - | - | - |
| 50-59 | 1.37 | 0.42 | 0.75-2.48 | 0.3 |
| 60-69 | 3.19 | 1.64 | 1.16-8.76 | 0.02** |
| 70-79 | 1.56 | 1.05 | 0.41-5.86 | 0.51 |
| 80+ | 1 |  |  |  |
| **Education** |  |  |  |  |
| No education | REF. | - | - | - |
| Standard 1-7 | 0.76 | 0.33 | 0.32-1.78 | 0.52 |
| Form 1-6 | 0.43 | 0.21 | 0.16-1.14 | 0.09* |
| Vocational training | 0.26 | 0.23 | 0.05-1.49 | 0.13 |
| University level + | 0.57 | 0.48 | 0.11-2.98 | 0.5 |
| **Employment** |  |  |  |  |
| Employed | REF. | - | - | - |
| Not working | 2.08 | 1 | 0.81-5.35 | 0.13 |
| Homemaker | 0.76 | 0.22 | 0.43-1.34 | 0.34 |
| **Marital Status** |  |  |  |  |
| Never Married | REF. | - |  | - |
| Separated & divorced | 2.48 | 2 | 0.51-12.13 | 0.26 |
| Widowed | 1.27 | 0.91 | 0.031-5.15 | 0.74 |
| Currently married/cohabitating | 1.84 | 1.19 | 0.52-6.50 | 0.34 |
|  |  |  |  |  |
|  |  |  |  |  |
| * | <0.1 |  |  |  |
| ** | <0.05 |  |  |  |
| *** | <0.01 |  |  |  |

Table 2. Logistic regression with key variables – Sexual Violence

|  |  |  |  |  |
| --- | --- | --- | --- | --- |
| **Sexual violence** | | | | |
| Characteristics | OR | Standard Error | 95% CI | P-value |
| **Sex** |  |  |  |  |
| Male | REF. | - | - | - |
| Female | 1.54 | 0.46 |  | 0.15 |
| **Age group** |  |  |  |  |
| 40-49 | REF. | - | - |  |
| 50-59 | 0.98 | 0.29 |  | 0.95 |
| 60-69 | 1.34 | 0.58 |  | 0.49 |
| 70-79 | 2.22 | 1.75 |  | 0.31 |
| 80+ | 1 |  |  |  |
| **Education** |  |  |  |  |
| No education | REF. | - | - | - |
| Standard 1-7 | 1.35 | 0.58 |  | 0.41 |
| Form 1-6 | 1.66 | 0.79 |  | 0.29 |
| Vocational training | 1 |  |  |  |
| University level + | 0.41 | 0.26 |  | 0.17 |
| **Employment** |  |  |  |  |
| Employed | REF. | - | - | - |
| Not working | 1.32 | 0.61 |  | 0.56 |
| Homemaker | 0.56 | 0.16 |  | 0.049** |
| **Marital Status** |  |  |  |  |
| Never Married | REF. | - |  | - |
| Separated & divorced | 3.04 | 2.27 |  | 0.14 |
| Widowed | 2.51 | 1.69 |  | 0.17 |
| Currently married/cohabitating | 2 | 1.13 |  | 0.21 |
|  |  |  |  |  |
|  |  |  |  |  |
| * | <0.1 |  |  |  |
| ** | <0.05 |  |  |  |
| *** | <0.01 |  |  |  |

Table 3. Logistic regression analysis with key variables, stratified by sex – Physical violence in Male

|  |  |  |  |  |
| --- | --- | --- | --- | --- |
| **Physical violence** | | | | |
| Characteristics | OR | Standard Error | 95% CI | P-value |
| **Age group** |  |  |  |  |
| 40-49 | REF. | - | - | - |
| 50-59 | 1.68 | 0.95 | 0.56-5.08 | 0.35 |
| 60-69 | 8.34 | 9.58 | 0,89-78.92 | 0.06* |
| 70-79 | 5.5 | 6.67 | 0.51-59.25 | 0.16 |
| 80+ | 1 |  |  |  |
| **Education** |  |  |  |  |
| No education | REF. | - | - | - |
| Standard 1-7 | 2.32 | 2.09 | 0.39-13.52 | 0.35 |
| Form 1-6 | 4.09 | 4.34 | 0.49-34.28 | 0.19 |
| Vocational training | 0.92 | 1.29 | 0.06-14.38 | 0.95 |
| University level + | 1.37 | 1.84 | 0.09-19.20 | 0.82 |
| **Employment** |  |  |  |  |
| Employed | REF. | - | - | - |
| Not working | 1.76 | 1.51 | 0.33-9.47 | 0.51 |
| Homemaker | 0.82 | 0.55 | 0.22-3.04 | 0.77 |
| **Marital Status** |  |  |  |  |
| Never Married | REF. | - |  | - |
| Separated & divorced | 1 |  |  |  |
| Widowed | 3.26 | 4.33 | 0.25-44.04 | 0.37 |
| Currently married/cohabitating | 10.68 | 11.19 | 1.37-83.32 | 0.02** |
|  |  |  |  |  |
|  |  |  |  |  |
| * | <0.1 |  |  |  |
| ** | <0.05 |  |  |  |
| *** | <0.01 |  |  |  |

Table 4. Logistic regression analysis with key variables, stratified by sex – Sexual violence in Male

|  |  |  |  |  |
| --- | --- | --- | --- | --- |
| **Sexual violence** | | | | |
| Characteristics | OR | Standard Error | 95% CI | P-value |
| **Age group** |  |  |  |  |
| 40-49 | REF. | - | - |  |
| 50-59 | 0.66 | 0.36 | 0.23-1.95 | 0.45 |
| 60-69 | 0.31 | 0.31 | 0.09-1.04 | 0.059* |
| 70-79 | 1 |  |  |  |
| 80+ | 1 |  |  |  |
| **Education** |  |  |  |  |
| No education | REF. | - | - | - |
| Standard 1-7 | 0.84 | 0.71 | 0.16-4.41 | 0.84 |
| Form 1-6 | 2.97 | 3.27 | 0.34-25.75 | 0.32 |
| Vocational training | 1 |  |  |  |
| University level + | 0.61 | 0.69 | 0.07-5.68 | 0.67 |
| **Employment** |  |  |  |  |
| Employed | REF. | - | - | - |
| Not working | 1.87 | 1.25 | 0.50-6.94 | 0.35 |
| Homemaker | 0.96 | 0.62 | 0.27-3.38 | 0.95 |
| **Marital Status** |  |  |  |  |
| Never Married | REF. | - |  | - |
| Separated & divorced | 2.46 | 3.75 | 0.12-48.99 | 0.56 |
| Widowed | 3.65 | 5.67 | 0.17-76.77 | 0.41 |
| Currently married/cohabitating | 2.59 | 2.95 | 0.28-24.25 | 0.41 |
|  |  |  |  |  |
|  |  |  |  |  |
| * | <0.1 |  |  |  |
| ** | <0.05 |  |  |  |
| *** | <0.01 |  |  |  |

Table 5. Logistic regression analysis with key variables, stratified by sex – Physical violence in Female

|  |  |  |  |  |
| --- | --- | --- | --- | --- |
| **Physical violence** | | | | |
| Characteristics | OR | Standard Error | 95% CI | P-value |
| **Age group** |  |  |  |  |
| 40-49 | REF. | - | - | - |
| 50-59 | 1.19 | 0.44 | 0.58-2.47 | 0.63 |
| 60-69 | 2.22 | 1.31 | 0.69-7.08 | 0.18 |
| 70-79 | 0.63 | 0.54 | .12-3.35 | 0.58 |
| 80+ | 1 |  |  |  |
| **Education** |  |  |  |  |
| No education | REF. | - | - | - |
| Standard 1-7 | 0.59 | 0.31 | 0.21-1.63 | 0.31 |
| Form 1-6 | 0.22 | 0.13 | 0.07-0.69 | 0.01** |
| Vocational training | 0.16 | 0.19 | 0.015-1.77 | 0.13 |
| University level + | 0.41 | 0.48 | 0.04-4.01 | 0.45 |
| **Employment** |  |  |  |  |
| Employed | REF. | - | - | - |
| Not working | 1.77 | 1.05 | 0.55-5.67 | 0.34 |
| Homemaker | 0.76 | 0.25 | 0.39-1.46 | 0.41 |
| **Marital Status** |  |  |  |  |
| Never Married | REF. | - |  | - |
| Separated & divorced | 0.93 | 1.08 | 0.09-9.12 | 0.95 |
| Widowed | 0.63 | 0.71 | 0.07-5.64 | 0.68 |
| Currently married/cohabitating | 0.72 | 0.76 | 0.09-5.74 | 0.75 |
|  |  |  |  |  |
|  |  |  |  |  |
| * | <0.1 |  |  |  |
| ** | <0.05 |  |  |  |
| *** | <0.01 |  |  |  |

Table 6. Logistic regression analysis with key variables, stratified by sex – Sexual violence in Female

|  |  |  |  |  |
| --- | --- | --- | --- | --- |
| **Sexual violence** | | | | |
| Characteristics | OR | Standard Error | 95% CI | P-value |
| **Age group** |  |  |  |  |
| 40-49 | REF. | - | - |  |
| 50-59 | 1.16 | 0.42 | 0.57-2.35 | 0.69 |
| 60-69 | 9.29 | 9.74 | 1.19-72.49 | 0.034** |
| 70-79 | 1.17 | 0.98 | 0.23-6.07 | 0.85 |
| 80+ | 1 |  |  |  |
| **Education** |  |  |  |  |
| No education | REF. | - | - | - |
| Standard 1-7 | 1.61 | 0.66 | 0.72-3.59 | 0.25 |
| Form 1-6 | 1.08 | 0.58 | 0.38-3.09 | 0.88 |
| Vocational training | 1 |  |  |  |
| University level + | 0.26 | 0.23 | 0.043-1.54 | 0.14 |
| **Employment** |  |  |  |  |
| Employed | REF. | - | - | - |
| Not working | 1.18 | 0.79 | 0.31-4.42 | 0.81 |
| Homemaker | 0.46 | 0.16 | 0.23-0.92 | 0.028** |
| **Marital Status** |  |  |  |  |
| Never Married | REF. | - |  | - |
| Separated & divorced | 3.36 | 2.92 | 0.62-18.36 | 0.16 |
| Widowed | 2.58 | 2 | 0.56-11.85 | 0.22 |
| Currently married/cohabitating | 2.29 | 1.51 | 0.63-8.35 | 0.21 |
|  |  |  |  |  |
|  |  |  |  |  |
| * | <0.1 |  |  |  |
| ** | <0.05 |  |  |  |
| *** | <0.01 |  |  |  |
